# **‘**They would rather not have known and me kept my mouth shut’: The role of neutralisation in responding to the disclosure of childhood sexual abuse

**DOI:** 10.1177/14733250221124300

**Published:** 2022-09-06

**Authors:** Claire Cunnington, Tom Clark

**Affiliations:** Department of Sociological Studies, 7315 University of Sheffield, Sheffield, UK; Department of Sociological Studies, 7315 University of Sheffield, Sheffield, UK

**Keywords:** sexual abuse, children, trauma, user research, mental health, adults, recovery, disclosure

## Abstract

There is a well-established literature examining how perpetrators of child sexual abuse (CSA) neutralise the norms and beliefs that ordinarily prohibit such behaviours. However, there has been substantially less focus on how such techniques of neutralisation might also be applied by people and groups who were not directly involved in the abuse, who we might expect to be more supportive. Drawing on a thematic analysis of an open-ended survey (n=140) and semi-structured interviews (n=21) with adults who experienced childhood sexual abuse this paper examines societal responses to disclosure. Identifying three key techniques of neutralisation, it explores how families, professionals and institutions use wider discourses that deny the victim/survivor, deny or minimise harm and silence by appealing to loyalty. The results demonstrate how significant others can constrain, rather than support, the process of disclosure and recovering from CSA.

## Introduction

Many people who have experienced childhood sexual abuse (CSA) will attempt to disclose the abuse at some point during the life-course. Unfortunately, there is a well-established evidence-base to suggest that the response to disclosure may not be helpful or supportive (Allnock and Miller, 2013; Livesey, 2002; Palmer et al., 1999). Indeed, there are a number of high profile cases to demonstrate that disclosure does not necessarily lead to action to counter the consequences of the abuse (Kavanagh et al., 2020; Lampard and Marsden, 2015; Mountjoy, 2019; Smith, 2016). Given the well-established impacts of CSA on physical and mental health (Maniglio, 2009), and the role of disclosure in potentially mitigating some of that trauma (see Collishaw et al., 2007; DuMont et al., 2007; Singh et al., 2013; Domhardt et al., 2015), there is a need for research that examines the characteristics of unsupportive or disbelieving responses to disclosure. More specifically, there is a paucity of research that highlights how attempts at disclosure can be dismissed or denied by people we might otherwise expect to be supportive and explores how those responses affect people who are attempting to disclose CSA.

This article presents the results of a study that examined how adults who were victims of sexual abuse as a child later experienced attempts at disclosure. The results suggest that family members, friends, professionals and wider society often reinforce silence by utilising a variety of ‘neutralisation techniques’ (Sykes and Matza, 1957). These techniques appear to reflect a deep-rooted social discourse that neither facilitates nor supports disclosure. In turn, this can constrain the capacity for recovery.

## Disclosure and childhood sexual abuse

The act of informing others about the experience of a traumatic event is usually referred to as disclosure. It is often identified as a key point of recovery for people who experienced sexual abuse as a child (Allnock and Miller, 2013). Of course, ‘recovery’ is ongoing process that is self-defined rather than a static point of arrival (Cunnington, 2020). Similarly, disclosure itself is not necessarily a ‘one off’ event and often is a lifelong experience that is repeated in a multitude of different relationships and contexts. Unfortunately, some responses to disclosure may not be supportive and might even constrain further attempts (Lamb and Edgar-Smith, 1994).

There are many barriers to disclosure. In a sample of 67 male and female survivors, Collin-Vézina et al. (2015) distinguish between three types of barriers. Firstly, there are those that occur from within the self, which include internalised victim blaming, self-protection mechanisms and immature development at time of abuse. Secondly, there are barriers that relate to others, including violence and dysfunction in the family, power dynamics, awareness of the impact of telling and fragile social networks. Finally, there are barriers that relate to the wider social world, including labelling, taboos around sexuality, a general lack of support services and wider cultural narratives about CSA.

Smith et al.*,* found that the majority of disclosures of CSA first occur when the individual is in their 20s or older, while 68% of 122 research participants in Jonzon and Lindblad (2015) first disclosed in adulthood. However, Kelly et al. (1991) reported that over half of their participants who reported childhood abuse had attempted to disclose the abuse at the time. Of these, only 5% were reported to statutory agencies. This suggests that when children disclose CSA, the response is frequently inadequate.

Indeed, Allnock and Miller (2013), also report that 42% of the disclosures their 60 participants made as children were not acted upon. Palmer et al. (1999) similarly found that in 384 cases of childhood disclosures to professionals, only 12% were acted upon. Therefore, one aspect of disclosure that requires further exploration is how CSA abuse disclosures are received and disrupted by people who we might otherwise expect to respond more supportively.

### Techniques of neutralisation

One explanation that may help to account for such poor responses to disclosure is ‘the theory of neutralisation’. This was first proposed by Sykes and Matza (1957) to explain how teenagers justify their delinquent behaviour in response to societal pressure to conform. They found five rationalisations: denial of responsibility, denial of harm, victim blaming, questioning or blaming authority and, finally, loyalty to the group. Collectively, these rationalisations minimise the guilt the individual feels about breaking a social norm.

Since Sykes and Matza’s landmark study, neutralisation has been applied to many deviant behaviours (see Maruna and Copes, 2005, for a review), including paedophilia. A study of 40 convicted child abusers, for example, found that they denied personal responsibility by blaming their behaviour on external issues, such as alcohol and marriage problems, whilst also denying harm to their child victims (Mann, 2012). Durkin and Bryant (1999) similarly found that the most common justification for abusing children was a lack of harm to the child. Child abusers also blamed the child for being ‘seductive’ or argued that they were defending the child’s right to sexual expression. The second most common justification was blaming authority by asserting that police, social workers and parents failed in their duty of care (Durkin and Bryant, 1999). Similarly, Copley (2014) reported sex traffickers blaming the child’s parents for involving their children in child sexual exploitation.

Denial of harm often involves the abuser objectifying the victim as not fully human, with the child merely existing for the gratification of the perpetrator (Finkelhor and Araji, 1986; Sykes and Matza, 1957). Thus, the child is denied status as an autonomous human who therefore cannot be conceptualised as a victim (O’Donohue et al., 2008; Quayle and Taylor, 2002).

Although not explicitly using the frame of neutralisation, Liotti (2017) examined how intra-familial abusers exploit familial loyalty. The child is torn between the natural desire to approach a parent for comfort and the equally natural imperative to flee from danger (Liotti, 2017). Solinski (2017) argues that there can be a state of ‘knowing and not knowing’ in families that often leads to a collusion of denial. This creates a situation where a child feels that failure to respond to their disclosure demonstrates that the abuse is acceptable, but the disclosure is not. This compounds the effects of abuse by making the act of disclosure shameful.

At a more organisational level, De Young (1988) has analysed how pro-paedophilia organisations attempt to represent their behaviour. Such groups typically argue that children should be free to express their sexuality with adults, thereby denying that CSA is harmful, and questioning whether abuse produces victims at all. This attempt to ‘reframe’ their activities is not necessarily limited to those who carry it out directly. Klein and Tolson (2015), assessed organisational responses to CSA in the Jerry Sandusky case, and found that Penn State University employed neutralisation techniques by describing the abuse as ‘horseplay’, denying responsibility for it, arguing that the victims had not been harmed, and finally, by appealing to the loyalty of staff to the organisation. Researchers have also identified negative organisational cultures in the Catholic church, social care, sporting organisations and educational establishments (Blakemore et al., 2017; Ferguson, 2007; Palmer and Feldman, 2017).

There are criticisms of neutralisation theory. Fritsche (2005) argues that the list of neutralisation techniques is arbitrary and that many aspects of the theory are accepted unquestioningly. For example, one key issue involves cause and effect. Fritsche (Fritsche, 2005) questions whether offenders overcome barriers to deviant behaviour before committing the abuse, or only afterwards as a post-facto rationalisation. However, while this would be difficult to determine in a research study, it also ignores the fact that all knowledge is constructed from the perspective of the knower in the moment. The key issue for researchers is, therefore, how people use their subjective positions to make sense of their own and others’ behaviour, and to examine how that informs their lifeworld (Maruna and Copes, 2005), regardless of when these rationalisations occur.

Alternate theories have examined perpetrators’ attempts to silence disclosure about their crimes. DARVO, for example, attempts to explain how perpetrators react to being confronted by their victims (Freyd, 1997). It lists three perpetrator responses: deny, attack, and reverse victim and offender. These actions can be compared to Sykes and Matza’s factors relating to denial and victim blaming. However, while DARVO has been effective in describing perpetrator behaviour it is less helpful in considering how disclosure is received and acted upon in wider contexts within the family, personal networks or professional organisations.

Therefore, this paper examines the experiences of people who have attempted to disclose CSA during their life-course and asks if they encountered ‘techniques of neutralisation’ during those attempts. More specifically, the paper asks the following questions:

• To what extent do adults who have experienced CSA in childhood encounter techniques of neutralisation when they attempt to disclose those experiences?
• How do those adults who have experienced sexual abuse as a child negotiate disclosure across their life-course?
• How have these experiences of neutralisation affected recovery?

In examining these issues, the paper explores how societal discourse can inhibit the process of disclosure. It seeks to understand how the family, professionals and institutions can fail to acknowledge the individual experience of child sexual abuse, and how such encounters can help to construct an over-arching tension between the individual need for disclosure and the social undesirability of discussing CSA.

## Methodology

The paper draws on results taken from a larger study that examined how adults who were subject to sexual abuse in childhood experience the lifelong process of recovery. Employing a qualitative sequential design (Creswell, 2013), an open-ended survey collected data from 140 participants, followed by 21 semi-structured interviews. Ethical approval for the research was granted by the research ethics board at the University of Sheffield and ethical practice was central to the research design and delivery. At each stage of data collection, all participants were given information sheets, reminded of their right to withdraw from the study at any point and asked for their written informed consent before completion. Taking a trauma-informed approach (Herman, 1992; Salter, 1995; Van der Kolk, 2014), the topics covered within the survey were clearly outlined on the cover page of the survey and accompanied by a statement that questions would not ask for any specific details of the abuse itself. Details of support services were also offered at each stage in case of distress.

**Figure 1.**
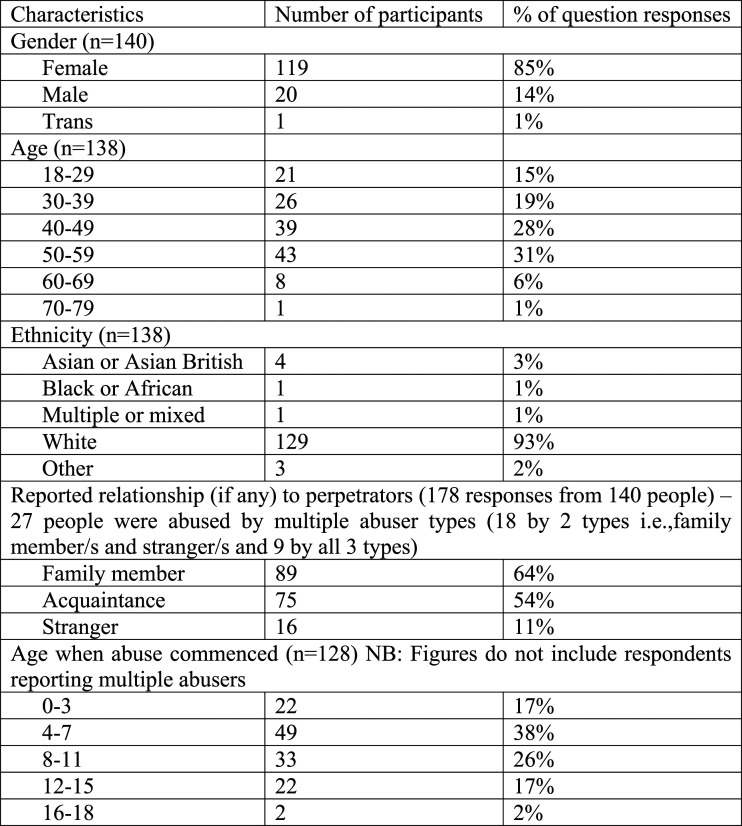
Characteristics of respondents.

Participants who self-identified as being ‘in recovery’ were purposively sampled through Twitter, Facebook and a research blog. The initial survey was also shared on websites and newsletters of organisations connected to CSA. The survey initially asked for some demographic information (see Figure 1), before examining more specific issues relating to CSA, drawn from an extensive literature review. This included questions of legal redress, medical treatment/counselling, relationships, creativity, religion/spirituality, careers, body care/exercise, campaigning/political activity, gender and identity.

Questions were phrased in an open-ended manner, for example:

• Have relationships with family, friends and others influenced your recovery? If so, how?
• Have legal actions, such as reporting the crime to the police, influenced your recovery? If so, how?

Demographic questions were assessed by calculating within item percentages. As no questions were compulsory, some items have missing data, while the item assessing ‘reported relationship to perpetrator’ records multiple responses.

Facilitated through NVivo11, data from the survey was subjected to the stages of thematic described by Braun and Clarke (2006). This systematic approach to qualitative analysis allows themes to emerge in vivo, while also allowing the researcher to pursue patterns across the data that reflect more theoretically positioned points of interest (Braun and Clarke, 2006). Analysis by the lead author utilised a six-stage process: familiarisation; initial coding; identifying themes; reviewing themes; defining themes; and evidencing themes.

For example, responses were initially coded to a node entitled ‘family’. As coding continued, it became clear that more granulation was required by the analysis. The responses discussing family of origin were, in the main, much more negative than those concerning later family members, such as husbands, wives and children. Thus, responses were further divided into family of origin and later familial relationships.

Disclosure emerged as a key part of negotiating CSA, with 99 survey respondents describing 258 specific examples of poor responses to disclosure or discussion of CSA. During the review stage of the analysis, it became clear that Matza and Sykes’ (1957) work on neutralisation could be used to further articulate how negative experiences of disclosure might be understood. More specifically, our attention was drawn toward an experiential tension between attempts to disclose, and the ways in which families, professionals and institutions can variously deny the victim/survivor, deny harm and ask for silence by appealing to loyalty or warning of negative consequences.

Although 93 of the survey respondents volunteered for interview, time constraints meant that 21 were selected for interview according to the principal of maximum variation (Quinn Patton, 2015). The dimensions for selection included responses that strongly reflected or challenged the over-arching themes identified in the survey (n=13); every 10th response (n=9); and a balance between the gender of the perpetrator (male=6; female=2). Emails were sent to all participants to thank them for their contribution, which also provided links to a website and email list where results would be disseminated.

Interview questions were designed to explore the emergent issues from the analysis of the open-ended survey data, which included the techniques of neutralisation. For example, the following questions explored the themes of medicine.

• What’s been good about the medical help you’ve had?
• What could be improved?
• Is there anything you wish they’d said or done?
• Is there anything missing from medical treatment?

Interview questions were provided beforehand on request and all interviews were carried out by the lead author in the medium chosen by the participant (face to face = 2; telephone = 5; Email, = 6; and Skype = 8). Participants were reminded of their right to withdraw from the study at any time and asked if they wanted to choose their own pseudonym. The interviews were recorded and again subjected to the process of thematic analysis.

Results are divided into four discrete sections that emerged during analysis. At each stage, careful consideration was given to whether the methods utilised were the most appropriate to ensure the trustworthiness of the research (Elo et al., 2014). In this respect, there was a great deal of congruence in the responses at both the open-ended survey and interview stages of the research, with very few dissenting opinions. For example, only one participant regarded abuse as ‘not particularly’ harmful in the survey, while the other 139 did experience it as harmful. Quotations represent and elucidate the opinions given by the majority of participants and basic quantitative details are given to indicate the levels of agreement.

## Results

### Blamed, ruined or recovering? Negotiating disclosure across the life-course

Nearly half (66) of the 140 comments in the survey concerning what *most* hindered the process of recovering were about poor reactions to talking about CSA. There was a clear tension in the results between the internal desire for the individual to disclose and an external, social pressure not to discuss CSA openly. In disclosing abuse, participants are attempting to create a safe and coherent environment within which they would like to exist. Ruth, for example, highlighted how talking about her experience of CSA enabled her to change her self-image from ‘victim’ to ‘survivor’:

‘I guess for me it's another step from me being a survivor from a victim being able to speak up, to be a little bit feisty, a little bit pushy and tell it like it is. It is something I get very passionate about.’

Unfortunately, participants suggested that there are significant external barriers to creating such an environment, including an over-arching familiarity with societal discourses of CSA. Participants worried that they would be viewed as tainted, non-functioning and permanently incapacitated members of society, as Agata explained when asked how society ‘talks’ about abuse:

‘Our lives are pictured as shells, trash, a cloth that can never be washed clean. It is unrealistic and hurtful. It creates a kind of a new fantastic beast – a ghost of a woman, who has been abused. The reality is there are millions of us and we talk to, work with, help, study with “non-abused” people every day.’

This influenced the types of emotion felt by participants in respect to CSA, with survey respondents variously reporting shame, a feeling of vulnerability, fear, anger, hate, disgust and sadness. 104 (out of 140) respondents suggested that society, in general, does not assist with recovery, as this anonymous survey respondent stated:

*‘*Society hinders recovery in SO many ways…The ubiquity of porn, the degradation of women in all types of media, make it clear that society is on our rapist's side - just as my family were on my father's side when I was being raped as a child. It feels the same, but on a bigger scale.’

Here, a clear line is drawn between the familial reactions to abuse and wider stigma concerning abuse, with judgemental attitudes towards CSA making it very difficult for survivors to reveal their experiences. This was explained by Sarah:

‘the focus is on the victim and what the victim did or didn't do, rather than the perpetrator.’ People [who have experienced CSA] are talked about as though they are always held in that position of being a child so that...we are infantilized a lot of the time.’

Disclosure risks the individual being both blamed for the abuse *and* seen as ‘ruined’ by it. It is unsurprising then that wider discourses around CSA affected their willingness to talk about abuse.

**Figure 2.**
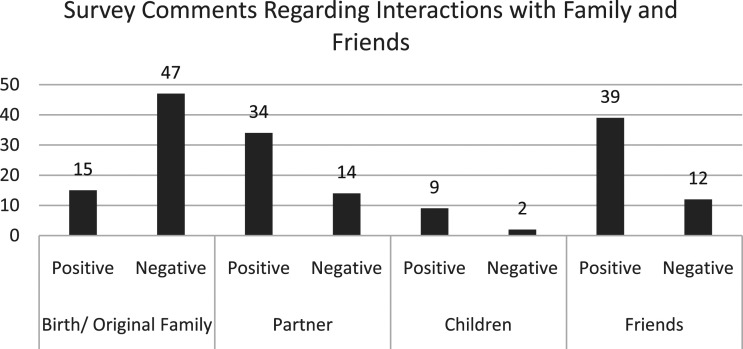
The influence of friends and family on recovering.

Interpersonal mechanisms also inhibited disclosure in interactions with family members, friends, partners and professionals. Figure 2 illustrates the frequency of survey comments regarding the influence that family and friends had on recovering from CSA.

33 survey respondents said that ‘family’ was *the* single factor most hindering their recovering, with nearly half (n=62) of survey respondents describing a poor family reaction to disclosing abuse. This was, in the main, relating to the individual’s family of origin. When this was explored further in interviews, 18 out of 21 interviewees reported that someone either knew the abuse was happening or had suspicions that it was taking place. 11 participants had clearly disclosed as children, and action was taken in only two of those cases to stop the abuse. In the other nine childhood disclosure cases, non-offending family members (6), police (1), school (1) or church (1) were aware of the abuse but took no effective action to stop it.

### Neutralising attempts at disclosure: higher loyalty, denial of the victim/survivor and denial of harm

The reported attempts at disclosure explored in the interviews indicate that, for many people, the abuse that they experienced was known about, but no one took any action. Instead, the disclosure was, in one way or another, suppressed. The techniques of neutralisation that participants described relate to three of those originally provided by Sykes and Matza (1957): an appeal to higher loyalty, denial of the victim/survivor and denial of harm.

### Appeal to higher loyalty

Appealing to higher loyalty is a technique of neutralisation that has been shown to be particularly effective when employed by a family member (Liotti, 2017). Ruth explained how her mother enabled the abuse she experienced from her father:

‘when I got there the mother was like ‘You need to go… whatever it is you have done to him, what it is you have fallen out with him about you need to go fix it because he's out in the garden burning his belongings on a bonfire and he's already beaten your brother up twice’ and the only way for me to placate him and stopping the beatings was for me to let him do whatever he wanted to do.’

Ruth thought that by ‘taking’ the abuse she was protecting her family from further violence. She was the sexual scapegoat for the family and her mother made a direct appeal to her familial loyalty. Unfortunately, exploitation of that loyalty did not stop there. One of the first institutions that a person who has been or is being abused might disclose to is via their primary care doctor or nurse. Ruth went on to detail how she was terrified that she would get pregnant with a ‘*mutant baby’* because of her father’s abuse and tried to tell her doctor, but her mother did not allow her to go in alone. She was further dissuaded from trying to disclose when her GP also appealed to her loyalty to the family:

‘the one time that the doctor actually looked at me and said to my face that ‘whatever was going on with me I needed to pull myself together and sort myself out because if I didn't my mother was going to end up in the loony bin for looking after me and I needed to stop being so bloody selfish.’’

She did not try to disclose the abuse again for years.

### Denial of the victim/survivor

Lynne explained that some of her family members were aware of the CSA because they were abused by the same person, her grandfather:

‘everybody knew, everybody had been abused by him as a child. It was his sisters, his brothers, his own children, you know, it was just everybody, but nobody talked about it, and you could sense that the blame was on the children or...you knew that they knew and that it was my fault’

Thus, some participants gave examples where families were extremely toxic, with adult family members maintaining this state by denying the victim/survivor – even when they were fully aware as they had also been a victim of the same perpetrator. Abuse is often viewed as shameful for the whole family, who are all blamed for it (Carter, 1993; McElvaney and Nixon, 2020). In response to this threat to family honour, family members may deny or minimise the abuse, defend the perpetrator and attack the victim/survivor. Again, Ruth explained:

‘My older brother and sister blamed me both for the abuse and for breaking up the family...Members of my extended family basically ostracized me because I had dragged the family name through the mud, and I had made everything public. I had destroyed the safe, quiet...they would rather not have known and me kept my mouth shut than actually report it and get it stopped.’

Her non-offending family viewed Ruth’s disclosure as *more* transgressive and disloyal than her father’s abusive behaviour.

Alongside the fact that family members can be the perpetrators of abuse, these examples demonstrate why the family of origin is reported as a negative arena for both disclosure and recovery (Figure 1). Three interviewees commented that cutting off all contact with their family was very significant in their recovering. Although one might assume that familial support *should* be crucial in recovering, it appears often to be the opposite: a cause of harm in and of itself.

As suggested by Ruth’s experience, in some cases the abuse is also ignored by professional organisations. Although most people who disclosed the abuse to police did so as an adult, two participants tried repeatedly as children. One survey respondent, who was abused by a family member and was also a victim/survivor of sexual exploitation, contacted the police on numerous occasions, but was treated as a criminal:

‘I was once asked to testify in court against a pimp when I was 13 and in return the police would investigate crimes I had reported. Once I had testified, I never heard from them again. Other members of staff in the care homes and secure units I grew up in wouldn’t even allow me to report abuse or sex crimes to the police because they didn’t believe me. I was sexually assaulted by a male police officer when I was 13 in front of his (female) colleague and she denied that it ever happened. I was also spat at by police officers, called derogatory names and laughed at while I was working on the streets in my preteens/early teens so have a huge distrust of the police.’

Despite being visible to police and social care as a victim of child sexual exploitation this survey participant was not supported, nor were her disclosures acted upon. This reflects the experiences of many children in the UK who experienced child sexual exploitation (Bedford et al., 2015; Jay, 2014)

Mary, who works as a senior practitioner in mental health services, saw further examples that resonated with her own experience of abuse. Here, she highlights how the behaviour of a child was used to define, and deny, their status as a victim/survivor:

‘I was at (hospital) sitting in behind a screen, one way screen with the trainees just watching a foster family, a family of a fostered child who had been sexually abused and the psychiatrist says, "Look how flirtatious that girl is," and I hadn't noticed how any flirtation whatsoever. I was really upset thinking, wondering if I was flirtatious without me knowing it because I kind of assumed he was right, and I was wrong…I look back at that and think he was wrong’

Mary felt that, in this case, the abuse victim was viewed as flirtatious and blamed for being seductive. This minimises the responsibility of the perpetrator, with the stigma also being shifted to the victim, who is denied her status as a victim. Participants gave further examples of how their status of victim/survivor was questioned in the course of, for example, giving evidence in court and negotiating a settlement with the Catholic Church.

### Denial of harm

According to Sykes and Matza (1957), denial of harm occurs where the perpetrator questions whether anyone was hurt by their behaviour and whether any real damage was done. However, this denial of harm can also extend to, and be perpetuated by, others. For example, one anonymous male survey respondent who was abused by a teenager was told by his family:

‘it wasn’t abuse. It was just experimenting.’

Fred similarly recounted his experience of a response from his GP that again appeared to deny the harm he had experienced:

‘I went to my GP and he basically said ‘25 years ago, well, it was a long time ago. Buddy, suck it up; get over it. I'm not surprised you've got bowel problems. Soldiers shit themselves on the battlefield."

Other people, the GP suggests, have it much worse, and Fred should, apparently ‘get over it.’

**Figure 3.**
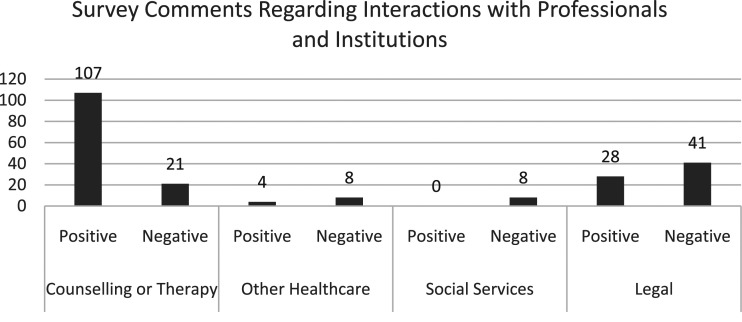
The influence of professionals and institutions on recovering.

Figure 3 shows survey comments regarding interactions with professionals and institutions, which will have included initial disclosure.

Evidently, the relative safety of counselling and therapy produced the most positive experiences. However, participants also described poor responses in those settings as well as from other groups, including social workers, teachers, doctors, police and church leaders. Thus, the dominant discourses *and* actions of society can be argued to reflect or reinforce the neutralising messages from the perpetrator(s), which is then reproduced within professional organisations and the individuals that exist within them.

## Discussion

This paper has examined how adults who have experienced sexual abuse as a child attempt to negotiate disclosure across their life-course, and the extent to which ‘techniques of neutralisation’ disrupt attempts to talk about that abuse. The use of such tactics by others may be deliberately silencing or a less conscious attempt to avoid a difficult conversation. Indeed, the findings demonstrate that there is, all-too-often, a mismatch between an individual’s desire to disclose and the hearer’s willingness to listen. These attempts are often understood to be negative in outcome, and potentially dangerous or harmful for individuals. While these responses may be intentional behaviours within family and professional contexts, they also result from tacit social pressures not to talk about CSA openly. All of which merely reinforces the feeling of danger in the individual, who is threatened with notions of ‘blame’ and ‘ruin’ rather than assisted with recovery.

In response to the first research question, many responses to disclosure by family, professionals and wider society do appear to be deliberate attempts to neutralise disclosure by variously denying the victim/survivor, denying harm or appealing to a higher authority. So, while some participants reported trying to tell someone as a child, be it family or support services, it appears that the majority of victims/survivors were only *heard* when they were adults – and even then, experiences of disclosure were often tacitly unsupportive. Therefore, participants describe what appears to be a society that can perpetuate ineffective and silencing responses to disclosure. These responses appear to be long-lived and enduring, intentional or tacit, but with the same underlying message to remain quiet.

Such neutralisation techniques and discourses could be challenged on multiple levels. Firstly, by public health messaging campaigns that aim to change community attitudes and prompt more effective responses to disclosures. Further to this, bystander intervention programs and campaigns could aim to increase the skills and capacity of the community to respond appropriately. Within applicable professions there should be an ongoing awareness raising campaign to educate and train staff to respond appropriately and effectively to disclosures of abuse. Appropriate family support should be offered that allows the open expression of feelings around this difficult and emotive subject but also centres and protects the victim/survivor from further harm.

In terms of the second research question, exploring disclosure over the life-course, there do seem to be differences in responses to people who disclose in childhood and adulthood. However, whenever a disclosure is made, there is always a risk of it being poorly received. Childhood disclosure appears more likely to be made to family members and ignored. This reflects other research. The National Society for the Prevention of Cruelty to Children (NSPCC), for example, has demonstrated that 66% of 60 children they interviewed had tried to tell someone at the time. They report that of 203 disclosures, only 117 (58%) were acted upon. With maternal disclosures, other research suggests that as few as 30% of mothers might take any action (Allnock and Miller, 2013).

Adult disclosures, on the other hand, are more likely to be made to friends or professionals than family (Jonzon and Lindblad, 2004; Lamb and Edgar-Smith, 1994). Again, however, responses can still be poor with the harm the abuse caused being minimised or denied completely. This has been explored more relating to institutional rather than individual responses. In a recent UK parliamentary survey of adult survivors (The APPG for Adult Survivors of Childhood Sexual Abuse, 2019), 40% of respondents stated that the police had not taken their initial reporting of CSA seriously (see also Gardner, 2012; Guerzoni and Graham, 2015; Lovett et al., 2018; Spraitz et al., 2017).

### Limitations

There are limitations to the present study. Firstly, the qualitative approach means that the research sought richness of data, not quantity of response. However, statistical generalisation was not the aim of the study. Instead, ‘information-rich’ participants were selected through a process of purposive sampling to provide detailed evidence of their experiences following CSA, particularly in terms of the negative reactions to disclosure. Therefore, the study does raise issues which deserve more extensive attention. Secondly, as the participants are now adults, they may be describing events that occurred many decades ago. However, it is worth highlighting that many participants drew a line from their childhood to their current experiences, describing a sense of continuity in terms of responses, relationships, professional services and discourses.

Demographically, the participants cannot be considered to be representative of the population. For example, the majority of participants identified themselves as female and white. Further study is required to establish if the experiences described above are found in other populations. This study is concerned with the perspective of those in recovery and does not, therefore, explore why significant others might resist attempts at disclosure, or even actively suppress them. It may be the case that they do not always realise they are acting in such a way. Further examination of such perspectives would be a very useful extension to the research base. Finally, it should also be noted that participants were self-selecting. This means that there is no secondary corroboration for the experiences or events they discuss. As self-selecting participants, the majority agreed with the premise of the study: that CSA causes harm. Thus, they do not reflect alternate opinions on that subject. Nevertheless, while the results presented here might not be applicable to all groups and contexts, there is little reason to suspect that they are not instructive with respect to the general constraints experienced by people attempting to disclose experiences of CSA. In these terms, and whilst further research is desirable, moderatum generalisations are possible (Williams, 2000).

## Conclusion

This article contributes to the field of CSA research by highlighting poor responses to disclosure of CSA and the use of neutralisation to silence the victim/survivor. By doing so, the paper aims to facilitate the process of addressing these discourses, improving services and assisting individuals in their recovery. Indeed, these findings are important because they demonstrate that the feelings, emotions and experiences associated with disclosure affect recovering. The participants in this research reported needs that included being heard, respected and valued as members of society. Recovering from CSA is not just influenced by personal interactions. An individual’s health and well-being are also affected by the stigma associated with abuse victims/survivors. Awareness of these factors need to be built into any plan to assist recovering from abuse. Van der Kolk and Najavits (2013) argue that treatment cannot ignore the interaction with others and that we ‘do things because we are a part of tribes, communities and groups’ (p.519). People who have experienced CSA are enmeshed in networks of individuals, interactions and institutions and it is beholden on all of us to consider how we respond to disclosure so that society supports, rather than hinders, recovering from CSA.
